# Optimizing the Substrate Uptake Rate of Solute Carriers

**DOI:** 10.3389/fphys.2022.817886

**Published:** 2022-02-03

**Authors:** Klaus Schicker, Clemens V. Farr, Danila Boytsov, Michael Freissmuth, Walter Sandtner

**Affiliations:** Center of Physiology and Pharmacology, Medical University of Vienna, Vienna, Austria

**Keywords:** solute carriers, kinetic model, optimization, evolution, secondary active transporters, substrate uptake

## Abstract

The diversity in solute carriers arose from evolutionary pressure. Here, we surmised that the adaptive search for optimizing the rate of substrate translocation was also shaped by the ambient extracellular and intracellular concentrations of substrate and co-substrate(s). We explored possible solutions by employing kinetic models, which were based on analytical expressions of the substrate uptake rate, that is, as a function of the microscopic rate constants used to parameterize the transport cycle. We obtained the defining terms for five reaction schemes with identical transport stoichiometry (i.e., Na^+^: substrate = 2:1). We then utilized an optimization algorithm to find the set of numeric values for the microscopic rate constants, which provided the largest value for the substrate uptake rate: The same optimized rate was achieved by different sets of numerical values for the microscopic rate constants. An in-depth analysis of these sets provided the following insights: (i) In the presence of a low extracellular substrate concentration, a transporter can only cycle at a high rate, if it has low values for both, the Michaelis–Menten constant (K_M_) for substrate and the maximal substrate uptake rate (V_max_). (ii) The opposite is true for a transporter operating at high extracellular substrate concentrations. (iii) Random order of substrate and co-substrate binding is superior to sequential order, if a transporter is to maintain a high rate of substrate uptake in the presence of accumulating intracellular substrate. Our kinetic models provide a framework to understand how and why the transport cycles of closely related transporters differ.

## Introduction

Cellular membranes are diffusion barriers for polar solutes. Uptake of these solutes into a cell or a subcellular compartment is, therefore, contingent on solute carriers (SLC). For this reason, SLCs are vital for many physiological functions. The latter include cellular uptake of nutrients and extrusion of toxic compounds from the interior of a cell (Hediger et al., 2004; Omote et al., 2006; Sano et al., 2020). In addition, SLCs are involved in higher order functions, such as neurotransmission (e.g., reuptake of neurotransmitters subsequent to their vesicular release; Rudnick and Sandtner, 2019; Bhat et al., 2021). Many of the SLCs can harvest the energy contained in the transmembrane ion gradients to drive uphill transport of their substrate against an opposing substrate gradient (Mitchell, 1979). These are termed concentrative or secondary active transporters, which either work as symporters or antiporters (Jennigs, 2018). Another class of SLCs only facilitates passive diffusion of a polar solute by providing an aqueous pathway, *via* which the solute can enter or leave the cell. The latter are termed facilitating or equilibrative transporters. Both, the concentrative and the equilibrative SLC operate by the alternate access mechanism (Jardetzky, 1966) which entails the following sequence of events: Extracellular substrate first binds to the transporter in its outward-facing conformation. On substrate binding the transporter rearranges to adopt the inward-facing conformation. From there the substrate is released into the cytosol. Subsequent to this, the carrier rearranges again to return to the substrate-free outward-facing conformation. From this point on, this series of reactions can repeat all over. Substrate uptake by a solute carrier is, therefore, a process, which encompasses several partial reactions. These include conformational change and binding/unbinding reactions of substrate and (co)-substrates to and from the transporter. These partial reactions form a closed loop, which is also referred to as the transport cycle.

We have recently described an approach to kinetic modeling of a solute carrier, which allows for deriving analytical expressions for its functional descriptors (Schicker et al., 2021). These include the K_M_ and the V_max_ for substrate uptake, the rate of basal substrate release from the interior of the cell, etc. The corresponding terms express these descriptors as a function of the microscopic rate constants used to parameterize the kinetic model. In the present study, we derived the defining terms for the substrate uptake rate of a sodium symporter for five different reactions scheme, which all adhere to the same transporter stoichiometry (Na^+^:substrate = 2:1). The rationale for obtaining these analytical terms was as follows: The substrate uptake rate is the only functional descriptor of a transporter, for which compelling arguments can be made that it has been optimized (i.e., maximized) by evolution. These are: the magnitude of solute flux through SLCs into a cell or a cell organelle is determined by the number of transporter units expressed on the cell or organelle surface and the substrate uptake rate (i.e., turnover rate) of the individual transporters. Accordingly, to maintain a substrate flux, which is commensurate with the physiological needs, the cell can either increase the number of transporters or the rate of substrate turnover. The former is associated with two problems: (i) Protein synthesis is energetically costly (Millward and Garlick, 1976; Waterlow et al., 1978; Siems et al., 1984) and (ii) additional transporters occupy space in the membrane. Membranes cannot be infinitely crowded by transmembrane proteins (Bar-Even et al., 2011). Having to have fewer transporters, thus, increases the energy efficiency of a cell/organism. This is expected to improve fitness at conditions in which nutrients are scarce. Accordingly, the substrate uptake rate of a solute carrier fulfills all criteria of a trait subject to evolutionary selection.

We emulated the evolutionary pressure on the substrate uptake rate by relying on an optimization algorithm. This searched for the set of microscopic rate constants, which returned the largest value for the substrate uptake rate at given intra- and extracellular concentrations of Na^+^ and substrate. The resulting sets of values provided by the optimization algorithm were subjected to an in-depth analysis. This analysis showed how a solute carrier must adjust its operation to cycle at a high rate at the various conditions/challenges, which it may encounter.

## Materials and Methods

### Numerical Simulations

**Figure 1:**
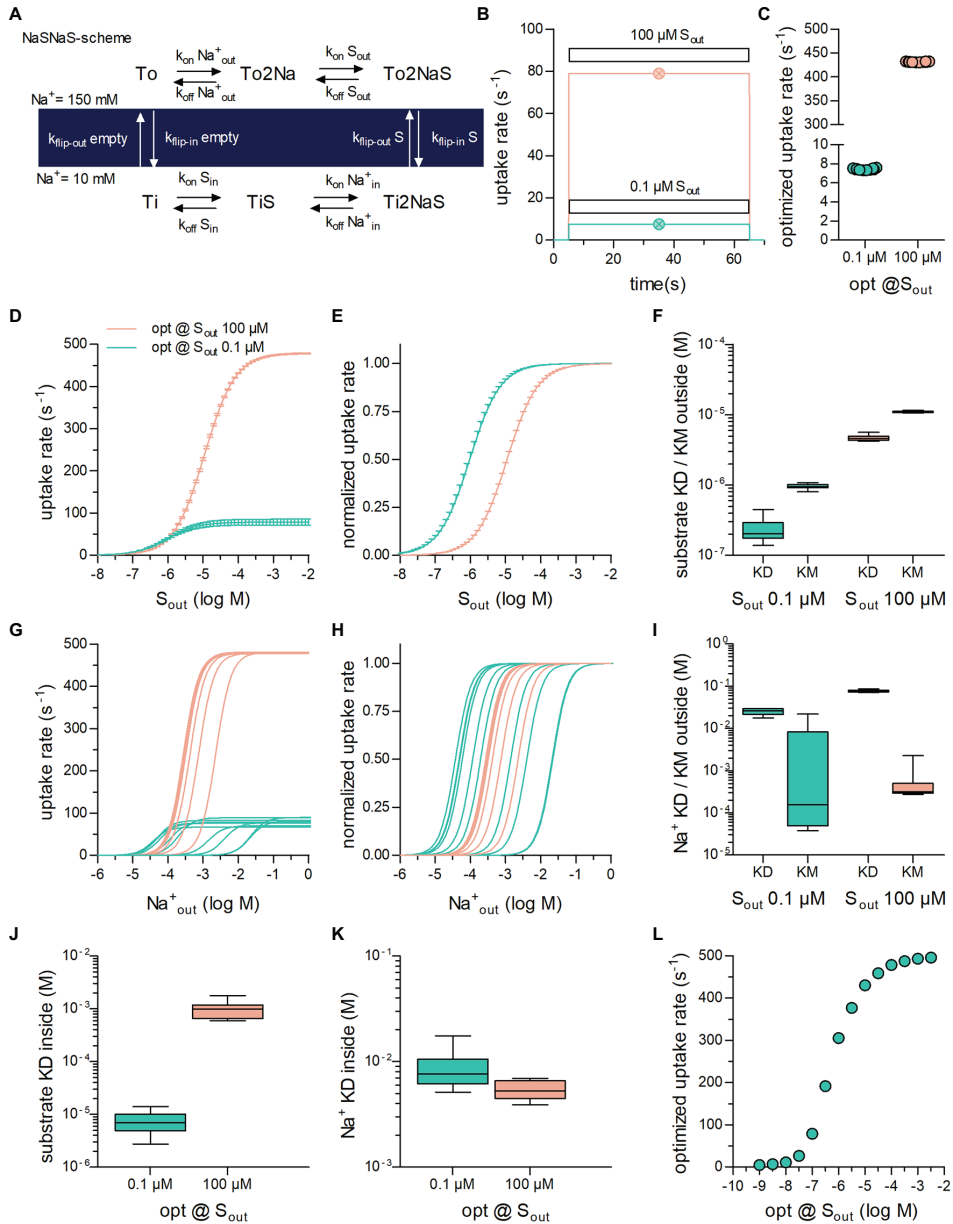
Optimization of secondary active transporters operating in a sequential binding mode for translocation of substrate at a low and high concentration. **(A)** Reaction scheme of a Na^+^ symporter in the sequential binding mode (“first in, first out”) referred to as NaSNaS: The apo-outward-facing transporter (To) first binds two Na^+^ ions (To2Na). On substrate binding, (To2NaS) the transporter rearranges to adopt the inward-facing conformation (Ti2NaS). Subsequent to the release of its cargo into the cytosol (TiNa2S → TiS → Ti) the substrate-free inward-facing transporter (Ti) undergoes a conformational change upon which the transporter returns to To. **(B)** Simulated substrate uptake rate of the symporter operating according to the scheme outlined in **(A)** after application of .1 μM (green trace) and 100 μM (magenta trace) of substrate (S_out_). For this simulation the microscopic rate constants (i.e., k_on_Na^+^_out_ and k_flip-in_S) were parameterized with the values for the optimized transporter T1 shown in Table 1. The symbols in green and in magenta show the substrate uptake rates computed for T1 with the analytical expression (see supplement) for .1 μM and 100 μM S_out_, respectively. The uptake rates obtained with the two different approaches were identical. **(C)** Substrate uptake rates of transporters optimized for .1 μM S_out_ (green open circles) and for 100 μM S_out_ (magenta open circles). The data points in the graph show the substrate uptake rates obtained from 10 optimization runs. The corresponding sets of numeric values for the microscopic rate constants are listed in Table 1. **(D)** The curves represent the substrate uptake rate as a function of S_out_ of transporters optimized for .1 μM S_out_ (in green) and 100 μM S_out_ (in magenta). The data are means from the 10 independently optimized transporters (T_1_–T_10_ and T_11_–T_20_), error bars indicate S.D. **(E)** The data in panel **(D)** were plotted as normalized values (V_max_ = 1). **(F)** Plotted are the K_D_ values for binding of the substrate to the outward-facing conformation of the transporter and the corresponding K_M_ values of transporters optimized for .1 μM and 100 μM S_out_, respectively. The coefficients of variation for the K_D_s were: .43 and .094 and for the K_M_s: .094 and .03 at .1 μM and 100 μM S_out_, respectively. **(G)** Substrate uptake rate as a function of ${Na}_{\mathrm{out}}^{+}$ of transporters optimized for .1 μM S_out_ (in green) and for 100 μM S_out_ (in magenta). The concentration dependence for ${Na}_{\mathrm{out}}^{+}$ differed considerably between transporters optimized for the same S_out_. **(H)** The same data as in **(G)** but normalized **(I)** Plotted are the K_D_ values for Na^+^ binding to the outward-facing conformation of the transporter and the corresponding K_M_ values of transporters optimized for .1 μM S_out_ and for 100 μM S_out_. The variation in the K_D_ values was much less than the variation in the K_M_ values. The coefficients of variation for the K_D_s were: .17 and .06 and for the K_M_s: 1.81 and 1.12 at .1 μM and 100 μM S_out_, respectively. **(J)** Shown are the K_D_ values of substrate binding to the inward-facing conformation of transporters optimized for .1 μM and 100 μM S_out_, respectively (coefficients of variation: .47 and .36). **(K)** Plotted are the K_D_ values of Na^+^ binding to the inward-facing conformation of transporters optimized for .1 μM S_out_ and for 100 μM S_out_ (coefficients of variation: .43 and .2). **(L)** Plotted are the optimized substrate uptake rates as a function of the S_out_, for which they were optimized. Each data point is the means ± SD of the substrate uptake rate obtained from 10 optimization runs. The optimized rate rose upon increase of S_out_. It leveled out at 500 s^−1^ because of the constraints imposed in the optimization.

Time-dependent changes in state occupancies of the model in Figure 1A were evaluated by numerical integration of the resulting system of differential equations using the Systems Biology Toolbox (Schmidt and Jirstrand, 2006) and MATLAB 2018a (MathWorks, Natick, MA, United States).

### Optimization of the Substrate Uptake Rate

Explicit expressions for the substrate uptake rate were derived as described previously (Burtscher et al., 2019; Schicker et al., 2021). Numerical sets of values for the microscopic rate constants maximizing these expressions were generated by a simulated annealing algorithm (Metropolis et al., 1953; Tsallis and Stariolo, 1996). In brief, in an initial step, a set of values for the microscopic rate constants was randomly drawn from independent normal distributions, centered at chosen start values with SDs of the same size. The substrate uptake rate for the drawn set was then calculated and compared with the substrate uptake rate calculated from the original set (i.e., the start values). The probability of accepting the new set was: prob. = exp.[−(−current Value + best Value)/*T*(iter)], with *T* being a temperature parameter, which was chosen to decrease exponentially with the number of iterations. If accepted, the new set was used for the next iteration, if not, the old set was retained. This procedure was repeated for 5,000 iterations. We safeguarded against trapping in a local maximum by reinitiating the algorithm with the maximum *T* value increased by 10% using the best parameter set found in the first round of iterations. If this yielded a better overall value for the substrate uptake rate, the next run was reinitiated with the original *T*. Otherwise *T* was increased by additional 10%. This procedure was repeated until reheating was unsuccessful in obtaining a better set of values for 10 times. On completion, the algorithm reported the best parameter set (i.e., the set of values which gave the largest substrate uptake rate).

In the optimization of the sequential binding order schemes, we kept the detailed balance constraint, by allowing the algorithm to vary all microscopic rate constants except one. This rate constant was then calculated from the other rate constants such that detailed balance was maintained. In the case of the random order binding scheme, it was necessary to calculate three rate constants because of the larger number of loops. Each set of microscopic rate constants was also evaluated for adherence to the other imposed constraints (e.g., diffusion limit for the association rates of substrate and co-substrate). Only if a set of values complied with the imposed constraints it was passed on to the annealing algorithm.

## Results

### Kinetic Models of SLC Can Predict Substrate Turnover Rates

Figure 1A shows the reaction scheme of a hypothetical symporter: In each cycle, the transporter translocates one substrate molecule through the membrane together with two Na^+^ ions. We selected this stoichiometry, because it is frequently observed: For instance, sodium-dependent glucose (SGLT1/SLC5A1 and SGLT2/SLC5A2; Wright et al., 2011) and phosphate transporters (PiT-1/SLC20A1 and PiT-2/SLC20A2; Forster et al., 2013) operate with this stoichiometry. For the sake of simplicity, we assumed binding of the two sodium ions to occur in a single reaction. In Figure 1B, we used this model to predict the rate of substrate uptake through the transporter by assuming that two different concentrations of extracellular substrate (S_out_) were applied, that is, .1 μM (magenta line in Figure 1B) and 100 μM (blue line in Figure 1B). As seen, on exposure of the cell to the substrate, the substrate uptake rate rose. The rise was large on application of 100 μM S_out_ and small on application of .1 μM S_out_. In the simulation, the substrate was removed after 60 s upon which the substrate uptake rate dropped to zero.

The data in Figure 1B were obtained by numerically solving the system of differential equations underlying the kinetic model. An alternative approach to compute the substrate uptake rate relies on deriving its defining analytical term. For the sake of space, we show the term in the supplement. Figure 1B also displays the substrate uptake rates obtained by this second approach in the presence of .1 μM (open circle in magenta) and 100 μM S_out_ (open circle in blue). It is evident that the substrate uptake rates predicted by the two methods were identical.

### Maximizing the Substrate Turnover Rate

The extracellular concentration of a substrate (S_out_) is a given quantity, that is, it is typically not subject to control by a single cell. Accordingly, SLC, which are tasked with transporting a substrate into the interior of a cell, must adjust their operation to the substrate concentration they encounter. The substrate concentration surrounding a cell can therefore, be assumed to exert evolutionary pressure. To emulate optimization of the substrate uptake rate by evolution, we maximized this rate, utilizing its defining function. For this purpose, we employed an optimization algorithm, which can approximate global minima/maxima of a function (i.e., simulated annealing—for details see the method section). The optimization algorithm can find the set of numeric values for the microscopic rate constants, which returns the largest value for the substrate uptake rate at given intra- and extracellular concentrations of Na^+^ and substrate.

We note that microscopic rate constants are *a priori* not mathematically constrained: They can assume values between zero and infinity. If they are permitted to vary across the entire mathematically possible range, the optimized substrate uptake rate will also adopt values between zero and infinity. It is a futile and meaningless exercise to maximize a function, for which it is known that no maximum exists. Fortunately, however, there are limits to the values of the microscopic rate constants. For instance, the association rates of co-substrates and substrate cannot be larger than the diffusion limit, which therefore imposes an upper limit on these rates. Likewise, the dissociation rates of substrate and co-substrate must also have an upper limit, because raising the dissociation rate constant results in affinity loss, which, when substantial, prevents the co-substrate and the substrate from interacting with the transporter in their physiological concentration ranges. It is also clear that a conformational change cannot occur with infinite velocity. We selected 1,000 s^−1^ as the upper limit for conformational transition rates. The choice of this value was based on information obtained from the literature (Zhang et al., 2007; Schicker et al., 2011; Hasenhuetl et al., 2018; Erdem et al., 2019). Another necessary constraint was to ensure that every set of optimized values complied with the rule of microscopic reversibility: The product of the rates in the forward direction in a loop must equal the product of the rates in the opposite direction. The combined constraints reshape the parameter space of the function, such that it harbors critical points, which do not exist in the unconstrained parameter space.

We performed ten optimization runs in which we assumed that S_out_ was .1 μM and 100 μM (Figure 1C). In all runs, we set the extra- and intracellular Na^+^ concentration to 150 mM and 10 mM and the intracellular substrate concentration (S_in_) to zero. The data points show the substrate uptake rates, to which the optimization algorithm converged when S_out_ was set to .1 μM (left column) and 100 μM (right column). The rates were low at .1 μM S_out_ (7.44 s^−1^ ± .08 s^−1^) and high at 100 μM S_out_ (431.40 s^−1^ ± .66 s^−1^). We emphasize that each point in Figure 1C represents a unique set of numeric values for the microscopic rate constants. In Table 1, we show the ten sets, which we obtained from the optimization runs where S_out_ was set to .1 μM and 100 μM. Although the values of the microscopic rate constants differed between sets, they all gave essentially the same substrate uptake rate when optimized for the same substrate concentration. We therefore conclude that the optimized rate can be realized by different sets of numeric values for the microscopic rate constants. Because of the large differences in these values, each set can be viewed to define a transporter with an individual phenotype. For this reason, we will from here on treat the term “transporter” and “a set of optimized values” as a synonymous description.

**Table 1:** Microscopic rate constants of optimized transporters.

S_out_ .1 μM	T_1_	T_2_	T_3_	T_4_	T_**5**_	T_6_	T_7_	T_8_	T_9_	T_10_
k_on_Na_out_ (M^−1^*s^−1^)	843.9	882.2	467824.2	172900	5103575	2668168	102801	11301.1	1305776	4117.5
k_off_Na_out_ (s−^1^)	18.4	23.1	13819.5	5033.6	89713.5	74711.5	2623.4	261	26895.4	121.7
k_on_Na_in_ (M^−1^*s^−1^)	144780.2	71436.7	3573458	63795.9	1053480	7485548	85572.9	242224.4	3001183	135554.6
k_off_Na_in_ (s^−1^)	1033.6	655.8	30079.9	757.1	5402.4	47223	516	1848.1	52802.7	562.9
k_on_S_out_ (M^−1^*s^−1^)	99756087	99321467	99900325	99781085	99971424	99749741	99780659	99786322	99766393	99715096
k_off_S_out_ (s^−1^)	23.9	19.1	13.8	17.6	41.4	17.4	25.2	21	44.7	19.6
k_on_S_in_ (M^−1^*s^−1^)	98320556	98934133	94504880	98557663	98674263	98891788	88619319	99302604	90444017	99325744
k_off_S_in_ (s^−1^)	578	688.3	661.2	299.1	966	859.6	1239.5	549	249.1	1091
k_flipin_S (s^−1^)	963	439.4	336	953.4	929	788.1	944.1	805	823	587.1
k_flipout_S (s^−1^)	887.5	623.2	630	910.1	368	816.3	924.2	910	802	263
k_flipin_empty (s^−1^)	942.8	966.1	877.3	818	689	649	635.4	549.2	877	951
k_flipout_empty (s^−1^)	332	307.5	399	272.4	136	266	201	217	190.5	384.3
**S_out_ 100 μM**	**T_11_**	**T_12_**	**T_13_**	**T_14_**	**T_15_**	**T_16_**	**T_17_**	**T_18_**	**T_19_**	**T_20_**
k_on_Na_out_ (M^−1^*s^−1^)	459612.4	464014.7	2147180	45080.1	13688.3	1819462	248255	112618	219098	1204854
k_off_Na_out_ (s^−1^)	34935.3	38332.5	154576.7	3414.9	1009.6	130362	19485.7	9815.8	16359.4	96032.4
k_on_Na_in_ (M^−1^*s^−1^)	4919690	3993649	12486840	6671406	20491995	16362423	6624730	12778355	30517371	11015465
k_off_Na_in_ (s^−1^)	25766.5	27738.4	59325	45413.9	79597.4	72486.4	35015.5	83510.1	137526	62251.8
k_on_S_out_ (M^−1^*s^−1^)	98615927	99737574	99830122	99892657	99369863	98985701	99041917	99525439	99582606	99961376
k_off_S_out_ (s^−1^)	518.2	484.7	517.8	505.8	523.3	485.6	415.6	378.9	486.2	463.8
k_on_S_in_ (M^−1^*s^−1^)	99322953	97562087	97649294	99729392	98979302	99787925	98616945	97467311	99781580	99521878
k_off_S_in_ (s^−1^)	107653.9	62043.6	114665.9	59904	177645	121233	87749	64743	118169.7	83413.3
k_flipin_S (s^−1^)	999.9	999.6	999.6	998.6	998.3	999.4	998.9	999.8	999.6	999
k_flipout_S (s^−1^)	994.9	942.8	984.3	993.2	977.2	990.2	998.5	992.2	917.3	965.9
k_flipin_empty (s^−1^)	982.7	980.8	999.8	962.7	964.8	954.5	961.3	987.6	962.8	938.2
k_flipout_empty (s^−1^)	998.5	999.9	998.4	999.6	999.2	998.9	999.4	999.2	999.9	998.9

The table shows ten sets of values for the microscopic rate constants optimized for .1 μM S_out_ (upper half) and ten sets optimized for 100 μM S_out_ (lower half). The depicted rate constants were obtained for the NaSNaS scheme. In these optimization runs, Na_out_, Na_in_, and S_in_ were set to 150 mM, 10 mM, and 0 μM, respectively. Values were rounded to one digit after the comma. The optimized substrate uptake rate was approximately the same within the two groups (i.e., 7.44 s^−1^ ± .08 s^−1^ and 431.40 s^−1^ ± .66 s^−1^ for .1 μM and 100 μM S_out_, respectively). This is in contrast to the values for the microscopic rate constants, which differed substantially even in the same group. Each set can be viewed to represent a transporter with an individual phenotype (T_1_–T_20_).

### Analysis of Optimized Transporters

The observations summarized in Table 1 warranted further scrutiny. They suggest that not all reactions, which a transporter undergoes, require the same extent of fine-tuning to support a high substrate uptake rate. We analyzed the transporters in Table 1 to understand, which reactions in the transport cycle do and do not require precise adjustment. Accordingly, we examined for each individual transporter, the values for a collection of descriptors of transporter function: (i) These included descriptors, which can be computed with the kinetic model but also obtained experimentally (i.e., V_max_ and the K_M_ for substrate/co-substrate) and (ii) descriptors, which can only be extracted from the kinetic model (i.e., K_D_s for substrate and co-substrate to the outward- and inward-facing conformation of the transporter). The rationale was as follows: If the values of a descriptor are all similar for transporters optimized for the same substrate concentration, we can conclude that fine-tuning of the reactions, which affect this descriptor, is essential for the realization of the optimized rate. Conversely, this is not the case, if the values are vastly different.

In Figure 1D, we plotted the substrate uptake rate as a function of S_out_ for the transporters in Table 1: The V_max_ values were low and high for the group of transporters, which were optimized for .1 μM and 100 μM S_out_, respectively. Within the two groups of transporters, the V_max_ values varied, but the magnitude of this variation was small: V_max_ of transporter optimized for .1 μM S_out_ and 100 μM S_out_, were 78.40 ± 6.61 s^−1^ and 478.38 ± 1.58 s^−1^, respectively. In Figure 1E, the data were normalized to maximum velocity, because differences in the apparent affinity of the optimized transporters for the substrate can be more readily appreciated in this representation. It is evident that the transporters, which were optimized for .1 μM S_out_ displayed a higher apparent affinity for the substrate than those optimized for 100 μM S_out_. The K_M_ values within groups fell into narrow ranges, that is, .95 ± .08 μM and 11.1 ± .31 μM for transporters optimized for .1 μM and 100 μM S_out_, respectively. Our analysis therefore indicates that transporters, must have low K_M_ and low V_max_ values to support a high substrate uptake rate when S_out_ is low, but a high K_M_ and a high V_max_ value when S_out_ is high.

We computed the K_D_ values for substrate binding to the outward-facing conformation of the individual transporters (Figure 1F): It is evident that the K_D_ values for the substrate were low and high for transporters, which were optimized for .1 μM and 100 μM S_out_, respectively (.1 μM S_out_: .24 μM ± .03 μM; 100 μM S_out_: 4.72 μM ± .42 μM). It also evident that the K_D_ values were smaller and that they covered a larger range than the corresponding K_M_ values (Figure 1F).

Inspection of Table 1 shows that the rate constants, which govern Na^+^ binding to the outward-facing state, are subject to large variations in transporters 1–10; in contrast, these rate constants differed substantially less in transporters 11–20, which were optimized to cope with 100 μM S_out_. We illustrated the resulting difference in apparent affinity for Na^+^ by plotting the absolute (Figure 1G) and normalized substrate uptake rate (Figure 1H) of the optimized transporters as function of the Na^+^ concentration: for those transporters, which were optimized for .1 μM S_out_ the apparent affinity for Na^+^ varied over five orders of magnitude (blue curves in Figures 1G,H). In contrast, for transporters optimized for 100 μM S_out_, the apparent affinity for Na^+^ fell into a narrow range (magenta curve in Figures 1G,H). Thus, if the extracellular substrate concentration is low, the Na^+^ binding reaction does not need to be fine-tuned to obtain optimal rates. However, at a higher substrate concentration, the Na^+^ binding reaction is subject to stringent constraints. This observation can be rationalized by taking into account that, in the optimization, Na^+^ was assumed to be present at a high concentration (i.e., 150 mM). At this concentration Na^+^ binding is unlikely to become rate limiting for substrate transport if S_out_ is low. At a higher substrate concentration, the apparent association rate of the substrate (k_app_) is expected to increase. In this scenario, Na^+^ binding becomes rate limiting, if it occurs at too low a rate.

We compared the K_D_ values for Na^+^ binding to the outward-facing conformation of the transporter *t* the corresponding K_M_ values (Figure 1I): Within each group, the variation in the K_D_ values was small, that is, K_D_ = 25.10 ± 3.91 mM and 79.21 ± 3.30 mM for transporters optimized for S_out_ .1 μM and S_out_ 100 μM, respectively. This contrasted with the large variation in the corresponding K_M_ values for Na^+^ seen for transporters optimized for .1 μM S_out_. This discrepancy can be explained as follows: The K_D_ values are determined by the ratio of the dissociation and association rates for Na^+^ but not by the absolute values of these rates. Conversely, given that the K_D_ values and the K_M_ values for Na^+^ were found to differ, it is safe to conclude that K_M_ values are highly dependent on the absolute values of these rates. These observations therefore imply that the affinity for Na^+^—rather than the velocity of Na^+^ binding to the transporter is subject to precise adjustment for supporting optimal uptake rates at low substrate concentrations.

Figures 1J,K summarize the K_D_ values for binding of substrate and of Na^+^ to the inward-facing conformation of the transporter, respectively. The coefficients of variation in K_D_ values of the inward-facing state were larger by a factor of 2 to 4 than the corresponding K_D_ values of the outward-facing state: K_D_S_in_ was 7.46 μM ± 3.4 μM and 701 μM ± 155 μM for transporters optimized for .1 μM S_out_ and for 100 μM S_out_, respectively. Likewise, the $K_{D}{Na}_{\mathrm{in}}^{+}$ was 8.81 mM ± 3.63 mM and 6.46 mM ± .70 mM for transporters optimized for .1 μM and 100 μM S_out_, respectively. The high K_D_S_in_ at 100 μM S_out_ was dictated by the low substrate affinity for the outward-facing state of transporters, which had been optimized for this condition, and the requirement to maintain microscopic reversibility. However, the larger variation in K_D_S_in_ and $K_{D}{Na}_{\mathrm{in}}^{+}$ indicates that the reactions, which define the substrate and co-substrate affinities for the inward-facing conformation, do not require as stringent an adjustment as those, which define the corresponding affinities to the outward-facing conformation. Finally, we surveyed transporter optimization over a large range of extracellular substrate concentration (1 nM to 10 mM; Figure 1L): The resulting optimized substrate uptake rate of the transporters increased as a function of S_out_ but leveled off at an uptake rate of about 500 s^−1^. This upper limit reflect the constraint imposed by the boundary conditions of the optimization (i.e., a diffusion-limited k_on_ and an upper limit of 1,000 s^−1^ for the rate of conformational transitions, see above). It was 249.4, 499.4, and 997.8 s^−1^ when the upper limit of the rate of conformational transitions was set to 500, 1,000, and 2,000 s^−1^, respectively.

### Testing Different Modes of Transport

**Figure 2:**
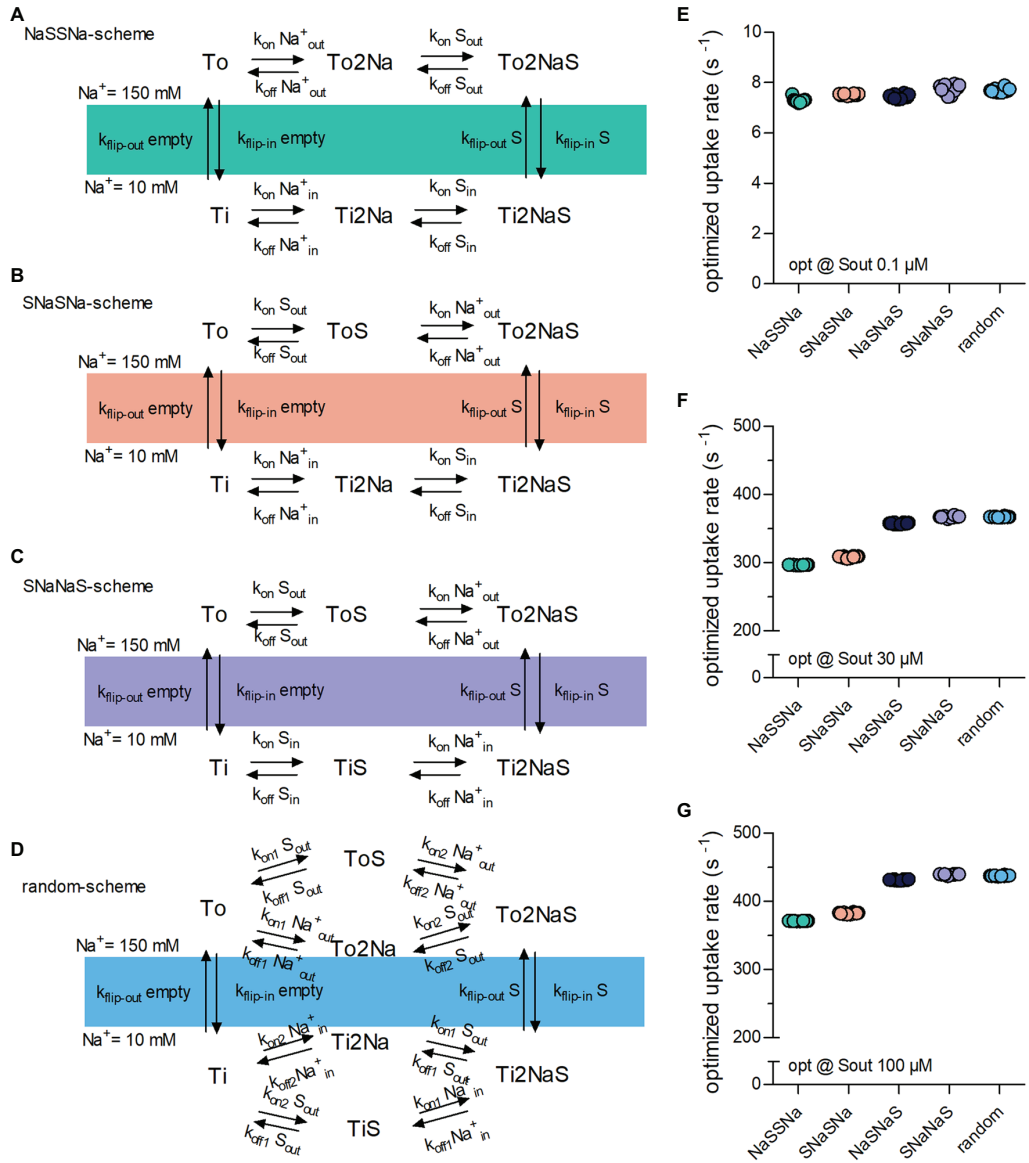
Binding order of co-substrate and substrate affects the optimized substrate uptake rate. **(A)** NaSSNa scheme (sequential). Na^+^ is the first to bind when the transporter adopts the outward-facing conformation and the last to dissociate from the inward-facing conformation. **(B)** SNaSNa scheme (sequential). Substrate is the first to bind to the outward-facing conformation and the first to dissociate from the inward-facing conformation. **(C)** SNaNaS scheme (sequential). Substrate is the first to bind to the outward-facing conformation and the last to dissociate from the inward-facing conformation **(D)**. Random binding order scheme. Na^+^ and substrate bind in random order. **(E)** Plotted is the optimized substrate uptake rate for all schemes of transporter optimized for .1 μM S_out_. At this low substrate concentration the optimized rate is approximately the same for all schemes. **(F)** Shown is the optimized substrate uptake rate for the different schemes optimized for 30 μM S_out_. The height of the optimized rate differed between schemes **(G)** the same as in **(F)** but of transporter optimized for 100 μM S_out_. The rank order of the optimized rates among schemes was the same as in **(F)**.

The reaction scheme in Figure 1A describes a hypothetical symporter, which binds co-substrate and substrate in a sequential order: The two Na^+^ ions are the first to bind when the transporter adopts the outward-facing conformation and the first to dissociate upon conversion of the transporter to the inward-facing conformation. In the subsequent description, we will refer to this reaction scheme as the NaSNaS scheme. This notation lists from left to right the order of the binding/unbinding events starting at the outward-facing apo-state (To) in clockwise direction. In Figures 2A–C, we show the reaction schemes for the three (sequential) alternatives. According to our notation, we refer to these as NaSSNa, SNaNaS and SNaSNa schemes. In addition, we also examined the reaction scheme of a transporter, in which the two Na^+^ ions and the substrate are allowed to bind in random order (Figure 2D).

We explored the impact of these five reaction schemes (Figures 1A, 2A–D) on the optimized substrate uptake rates by raising S_out_ from .1 (Figure 2E) to 30 (Figure 2F) and 100 μM (Figure 2G) and by conducting 10 optimization runs for each reaction scheme. As evident from Figure 2E, the substrate uptake rate was roughly the same for all schemes, when S_out_ was low (i.e., .1 μM). However, when optimized for a higher S_out_, the schemes differed in the magnitude of the optimized substrate uptake rates, which they were able to support. The rank order was as follows: NaSSNa < SNaSNa < SNaSNa < SNaNaS = random. The rank order was the same for transporters optimized for 30 μM and 100 μM S_out_, (*cf*. Figures 2F,G). Thus, random order of substrate and co-substrate binding and SNaNaS are best suited to support a large substrate uptake rate.

### Raising the Intracellular Substrate Concentration

Due to the way SLC operate, the intracellular concentration of the substrate and the substrate uptake rate are inversely correlated. This can be explained as follows: The substrate must be released into the cytosol to complete a full cycle. As the intracellular substrate concentration (S_in_) increases progressively during uptake, rebinding of the substrate to the inward-facing conformation occurs at a more frequent rate. This hampers progression through the transport cycle and thus reduces the substrate uptake rate.

Here we propose that—similar to the extracellular substrate concentration (S_out_)—S_in_ can also exert an evolutionary pressure on the operation of a solute carrier. Depending on the physiological context, transporters may encounter intracellular concentrations of their cognate substrate, which range from low to high levels. This can be illustrated by two examples in the SLC6 family: The cytosolic concentrations of the monoamines dopamine, norepinephrine, and dopamine are expected to be low. This is because of the presence of vesicular monoamine transporters (vMAT1/SLC18A1 & vMAT2/SLC18A2), which shuffle cytosolic monoamines into vesicles (Yaffe et al., 2018). Accordingly, under physiological conditions, monoamine transporters for dopamine (DAT/SLC6A3), norepinephrine (NET/SLC6A2) and (SERT/SLC6A4) are unlikely to encounter high intracellular concentrations of their substrate. Conversely, the creatine transporter-1 (SLC6A8) must maintain substrate influx in the presence of millimolar intracellular creatine (Snow and Murphy, 2001). It is clear that a solute carrier, which must support a high substrate uptake rate against high S_in_, must adjust its operation differently than a transporter, which does not need to overcome the hurdle imposed by frequent rebinding of the substrate to the inward-facing conformation.

**Figure 3:**
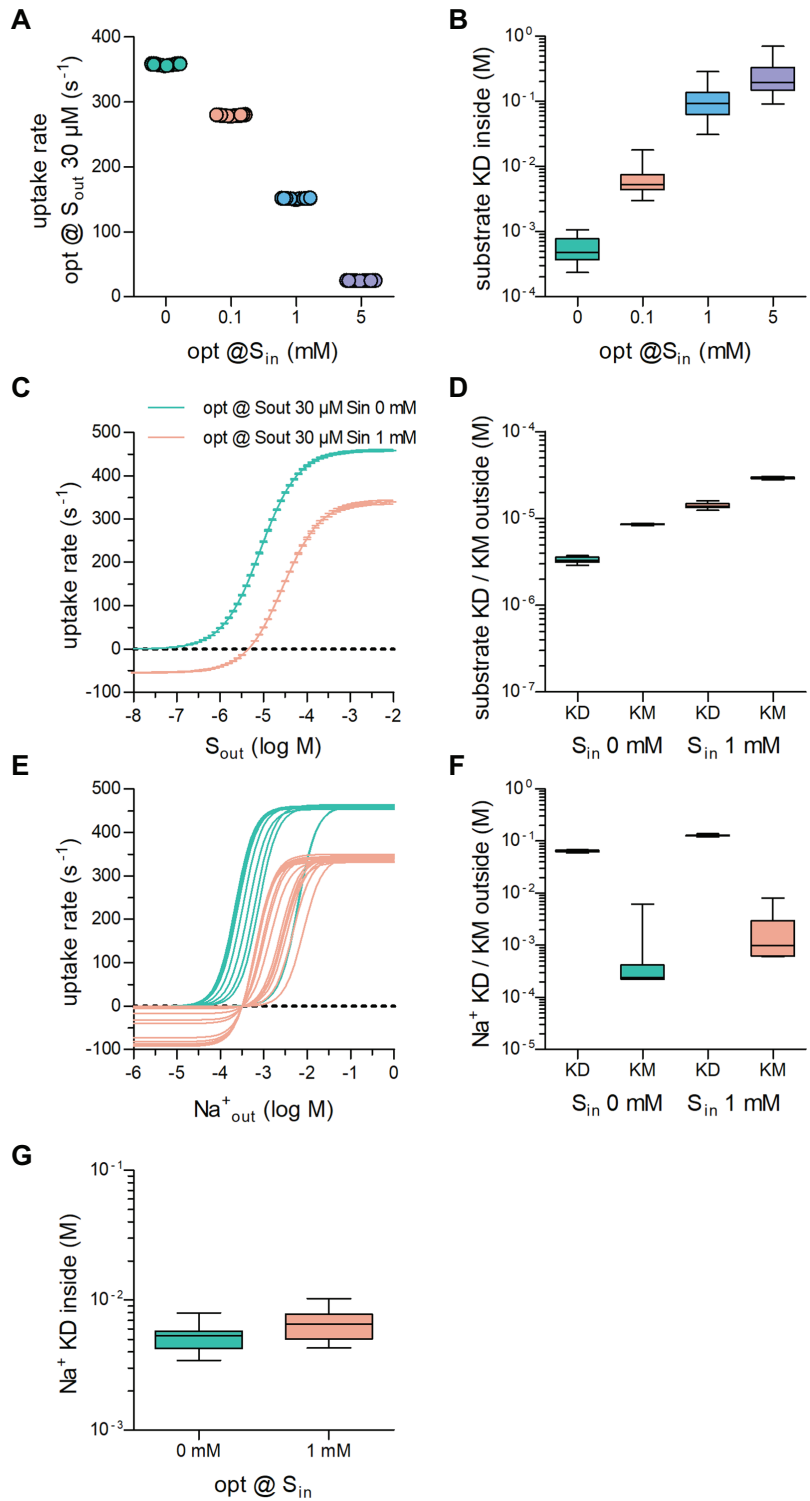
The optimized substrate uptake rate depends on the intracellular substrate concentration (S_in_). **(A)** Plotted are the optimized substrate uptake rates for transporters operating according to the NaSNaS scheme. S_out_ was set to 30 μM in all optimization runs. The data show the substrate uptake rate of transporters optimized for 0 mM, .1 mM, 1 mM, and 5 mM S_in_, respectively. The rate decreased with rising S_in_. Twenty optimization runs were carried out for each condition. **(B)** Shown is the K_D_ of substrate binding to the inward-facing conformation of transporters in **(A)**. The K_D_ decreased as S_in_ increased. **(C)** The curves show the substrate uptake rate as a function of S_out_ of transporters optimized for 0 mM (green) and 1 mM S_in_ (magenta). In the presence of S_in_, the substrate uptake rate assumed negative values when S_out_ was low. In this range of S_out_, the transporters cycled in the reverse direction. In the presence of 1 mM S_in,_ V_max_ was reduced. **(D)** Plotted are the K_D_ values for substrate binding to the outward-facing conformation of the transporter and the corresponding K_M_ values of transporters optimized for 0 mM S_in_ and for 1 mM S_in_. At high S_in_ both the K_M_ and the K_D_ values rose. The coefficients of variation for the K_D_s were .080 and .070 and for the K_M_s .020 and .026 at .1 μM and 100 μM S_out_, respectively. **(E)** Shown is the concentration dependence of the substrate uptake rate for ${Na}_{\mathrm{out}}^{+}$ of transporters optimized for 0 mM S_in_ and 1 mM S_in_. The dependence on the Na^+^ concentration was highly variable between transporters optimized for the same S_in_. **(F)** Plotted are the K_D_ values for Na^+^ binding to the outward-facing conformation of the transporter and the corresponding K_M_ values of transporters optimized for 0 mM S_in_ and for 1 mM S_in_. The variation in the K_M_ values was larger than the variation in the K_D_ values. The coefficients of variation for the K_D_s were .041 and .033 and for the K_M_s 2.03 and .97 at .1 μM and 100 μM S_out_, respectively. At high S_in_ both the K_M_ and the K_D_ values for Na^+^ increased. **(G)** KDs for Na^+^ for the inward facing conformation of the transporters optimized for 0 mM and 1 mM S_in_, respectively.

We first optimized the substrate uptake rate of a transporter operating according to the NaSNaS scheme (illustrated in Figure 1A) by performing 20 optimization runs each, where S_out_ was 30 μM and S_in_ was set at 0, .1, 1, and 5 mM. It is evident from Figure 3A that the optimized substrate uptake rate decreased by raising S_in_. This is in line with the inverse correlation of S_in_ and the substrate uptake rate discussed above. Figure 3B illustrates the range of K_D_ values for substrate binding to the inward-facing conformation of the transporters, which had been optimized to cope with different concentrations of S_in_: K_D_ values of substrate for the inward-facing conformation increased as S_in_ was raised. This was to be expected, because lowering the intracellular affinity for substrate reduces the extent by which S_in_ can rebind, and it thus allows for a higher substrate uptake rate in the presence of S_in_.

We then compared the uptake rate of transporters optimized for 30 μM S_out_ and 0 mM or 1 mM S_in_ over a large range of extracellular substrate concentration. As can be seen from Figure 3C, it was inevitable that transporters optimized in the presence of 1 mM S_in_ had negative uptake rates at low extracellular substrate concentrations, that is, the transporters cycled in the backward rather than the forward mode and hence mediated substrate efflux from the cell. It is also clear that the presence of 1 mM S_in_ reduced the maximum achievable uptake rate V_max_ in the forward transport mode and shifted the K_M_. Because K_M_ and K_D_ differ (*cf*. Figure 1F), we examined the range of K_D_ values for substrate binding to the outward-facing conformation of transporters optimized for 0 and 1 mM S_in_; these are illustrated together with the corresponding K_M_ values in Figure 3D: Both the K_D_ values and the K_M_ values for substrate increased, if the transporter had to cope with a high intracellular substrate concentration. The variation in these parameters was low (coefficient of variations = .070 and .025 for K_D_ and K_M_, respectively, of transporters optimized in the presence of 30 μM S_out_ and 1 mM S_in_). We conclude that the decrease in the apparent (K_M_) and the true affinity (K_D_) for the substrate, is required to allow for rapid cycling of the transporters in the presence of high S_in_.

Finally, we examined how the selective pressure exerted by high intracellular substrate affected the affinity of the transporters to the co-substrate ion. As can be seen from Figure 3E, many different solutions emerged: On average, transporters optimized to cope with 1 mM S_in_ (magenta lines in Figure 3E) required higher extracellular Na^+^ concentrations to support substrate uptake than those optimized in the absence of intracellular substrate (green lines in Figure 3E). However, in both groups, the optimized transporters displayed highly variable responses to ${Na}_{\mathrm{out}}^{+}$ . This is reflected in the large range of the K_M_ values for Na^+^ (Figure 3F). In contrast, the K_D_ of Na^+^ for binding the outward-facing conformation did not vary to any substantial extent. This observation is consistent with our conclusion from Figure 1I, namely, that the constraint is imposed by the true affinity for Na^+^ rather than by its association rate (see above). We also computed K_D_s for Na^+^ binding to the inward-facing conformation of the transporters optimized for 0 mM and 1 mM S_in_: There was an overlap in the range of K_D_ values for ${Na}_{\mathrm{in}}^{+}$ (Figure 3G). In addition, their variation was larger than that of K_D_ values for ${Na}_{\mathrm{iout}}^{+}$ (*cf*. Figures 3F,G). Hence, we conclude that Na^+^ binding to the inward-facing conformation need not be stringently adjusted to allow for high substrate uptake rates.

### Binding Order Affects the Magnitude of the Optimized Substrate Uptake Rate Also in the Presence of S_in_

**Figure 4:**
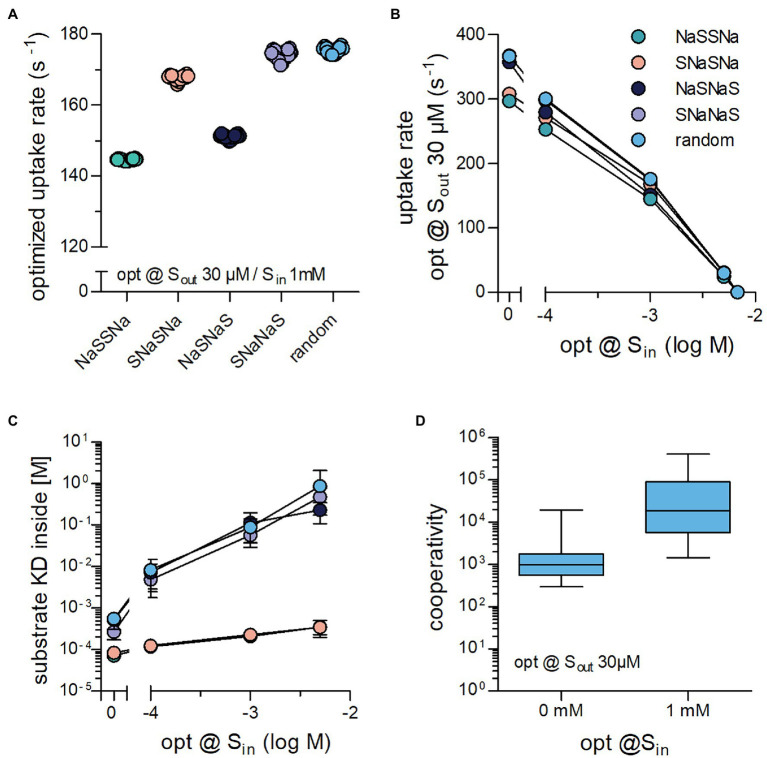
Binding order of co-substrate and substrate affects the optimized substrate uptake rate, when S_in_ is high. **(A)** Shown are the substrate uptake rates for transporters, which operate according to the reaction schemes illustrated in Figures 1A, 2A–D and which were optimized for 30 μM S_out_ and 1 mM S_in_. The optimized substrate uptake rate differed between reaction schemes. The data are the rates obtained from 20 optimization runs. **(B)** Plotted are the substrate uptake rates for all reaction schemes optimized for 0 mM, .1 mM, 1 mM, 5 mM, and 6.75 mM S_in_, respectively. The data are means ± SD of the substrate uptake rates obtained from 20 optimization runs. At 6.75 mM S_in_, the substrate uptake rate was zero for all reaction schemes. This is due to the fact that the concentrative power is defined by the transport stoichiometry (Na^+^: substrate = 2:1) and the Na^+^ gradient rather than the reaction scheme: With a gradient of 150 mM external to 10 mM internal Na^+^, the concentrative power of the transporters is 225, which yields 6.75 mM S_i_ at 30 μM S_out_ **(C)** Shown are K_D_ values for the substrate binding to the inward-facing conformation for all reaction schemes of transporters optimized to 0 mM, .1 mM, 1 mM, and 5 mM S_in_, respectively. While the absolute values of this K_D_ differed between schemes, they all rose with increasing S_in_. **(D)** The random binding order scheme, allows for Na^+^ and substrate to bind in a cooperative manner: The affinity for substrate is high when Na^+^ is bound and low in its absence. Cooperativity was defined as the ratio of the K_D_ of substrate binding to the inward-facing conformation in the absence and presence of Na^+^. Transporters optimized for 1 mM S_in_ displayed larger cooperativity values than those optimized for 0 mM S_in_ (*p* < .0001; Wilcoxon signed rank test).

We next addressed the question, if the binding order of substrate and co-substrate determined the cycle rate of a transporter challenged with high concentrations of intracellular substrate. Optimization runs were carried out with S_out_ = 30 μM and S_in_ = 1 mM for all five schemes (*cf*. Figures 1A, 2A). It is evident from Figure 4A that these schemes differed in the magnitude of the optimized substrate uptake rates, which they were able to support. The rank order was NaSSNa < NaSNaS < SNaSNa < SNaNaS = random. This rank order differed from that observed in the absence of S_in_ (*cf*. Figure 2F). At constant S_out_ = 30 μM, we also varied the internal substrate concentration by lowering to S_in_ 100 μM and raising it to the point, where net uptake rat was zero (Figure 4B). For all schemes we found an optimized substrate uptake rate of zero when S_in_ was 6.75 mM. This was to be expected because at the chosen concentrations of Na^+^ (i.e., 150 mM ${Na}_{\mathrm{out}}^{+}$ and 10 mM ${Na}_{\mathrm{in}}^{+}$) the concentrative power (S_in_/S_out_) of the transporter is 225. This numerical value is identical for all schemes, because they are governed by the same transport stoichiometry. Accordingly, the transporters cannot further cycle productively in a forward direction, when S_in_ becomes 225 times larger than S_out_ (30 μM * 225 = 6.75 mM). For all schemes, we extracted the K_D_ of substrate binding to the inward-facing conformation of the transporters optimized at varying S_in_ (Figure 4C). In all instances, this K_D_ increased with increasing S_in_. However, the magnitude of the drop in affinity depended on the reaction scheme.

For several secondary active transporters, binding of substrate and co-substrate was shown to occur in a cooperative manner: The apparent substrate affinity for the transporter depended on the concentration of the co-substrate (Meinild and Forster, 2012; Perez et al., 2014; Hasenhuetl et al., 2018; Erdem et al., 2019). It was low and high when the concentration of the co-substrate was low and high, respectively. Thus, the substrate can bind with higher affinity to transporters, when they are bound to the co-substrate (e.g., Na^+^). In this way, the concentration of the co-substrate determines the abundance of high and low affinity states for the substrate. Under physiological conditions, the co-substrate concentration is lower on the intracellular than on the extracellular side. Accordingly, cooperative binding is predicted to promote the forward cycling mode by reducing the substrate affinity to the inward-facing conformation. In fact, the drop in intracellular affinity resulting from cooperative binding is a requirement for maintaining a large substrate uptake rate at high S_in_ (Erdem et al., 2019). Notably, cooperative binding is contingent on a random binding order for substrate and co-substrate. For this reason cooperative binding can only be assessed in the random binding order scheme. Based on this consideration, a rise in S_in_ is predicted to increase the extent of cooperativity. We verified this prediction in optimization runs and extracted the cooperativity for transporters optimized at 30 μM S_out_ and 0 mM S_in_ or 1 mM S_in_ by calculating the ratio K_D_S_in_ in the absence of bound Na^+^/K_D_Si_n_ in the presence of bound Na^+^. It is evident from Figure 4D that there is a large range of optimized solutions, but on average cooperativity was more pronounced at higher S_in_.

### Substrate Selectivity Increases the Substrate Uptake Rate

**Figure 5:**
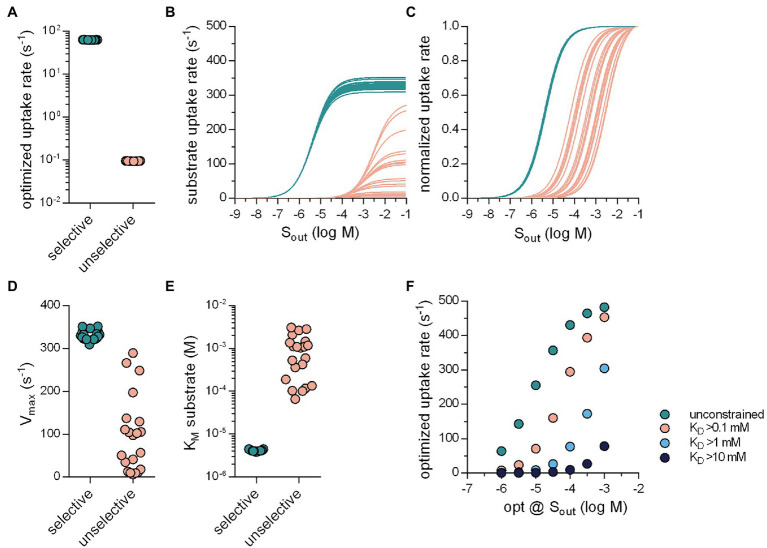
Selective transporters can support larger substrate uptake rates. **(A)** Optimized substrate uptake rates for selective (green circles; 63.79 ± .19 s^−1^; *n* = 20) and unselective transporters (pink circles; .095 ± .0006 s^−1^; *n* = 20). The transporters were optimized for 1 μM S_out_. The substrate K_D_ of the unselective transporters was not allowed to drop below 10 mM. For the optimizations we used the random binding order scheme. **(B)** Substrate uptake rate as a function of S_out_ for the selective (green lines) and the unselective transporters (pink lines). **(C)** The same data as in **(B)** but normalized **(D)** V_max_ values of selective and unselective transporters. The coefficient of variation of the V_max_ values was .034 and .91 for selective and unselective transporters, respectively **(E)** K_M_ values of selective and unselective transporters. The coefficient of variation of the K_M_ values was .042 and .90 for the selective and unselective transporters, respectively. **(F)** The substrate uptake rate as a function of the concentrations of S_out_ for which these rates were optimized. The green circles are the optimized rates of the selective transporters. The circles in pink, light blue, and dark blue are the optimized rates of unselective transporters, for which the substrate K_D_ was not allowed to drop below .1 mM, 1 mM, and 10 mM, respectively (*n* = 10 for each S_out_). The optimized rates of the unselective transporters were lower than that of the selective ones.

Evolution also optimized SLCs for substrate specificity. Selective transporters presumably arose from unselective ancestors. As a starting point, we posited that an unselective SLC must display low affinity for the various substrates: It is difficult to envisage a substrate binding site, which can provide strong bonding interactions to accommodate many distinct molecular scaffolds. We also assumed that, in the evolutionary trajectory from an unselective to a specific transporter, an increase in substrate specificity ought to translate in higher uptake rates for the substrate. Accordingly, in the optimization, we modeled an unselective solute carrier as a transporter, which had a low (true) affinity (i.e., a high K_D_) for substrate by implementing a constraint, which prevented the substrate K_D_ from dropping below a user-defined arbitrary value (e.g., 10 mM). With this constraint in place, the optimization algorithm only returned sets of values for the microscopic rate constants, which defined transporters with a high K_D_ for substrate. In the subsequent description, we refer to such sets as unselective transporters. In contrast, sets generated in optimization runs, in which the K_D_ for substrate was not constrained, are referred to as selective transporters. For the optimizations summarized in Figure 5, we employed the random binding order scheme, we assumed zero-trans conditions and the presence of 1 μM S_out_. Figure 5A shows the result for 20 selective and unselective transporters (K_D_ ≥ 10 mM): It is evident that the optimized substrate uptake rat of the unselective transporters (magenta symbols, Figure 5A) was lower by about three orders of magnitude than that of the selective SLCs (green symbols, Figure 5A). In Figure 5B, we examined the Michaelis–Menten kinetics of the substrate uptake rate of these optimized transporters. Figure 5C shows the same data normalized to V_max_ to illustrate the distribution of K_M_. The V_max_ values of the unselective transporters were lower than those of the selective SLCs but they varied over about orders of magnitude (Figure 5D). Similarly, the K_M_ values of the unselective transporters, which were consistently higher than those of the selective SLCs, were again highly variable. In Figure 5E, we show optimized rates as a function of the concentration of substrate, for which the rates were optimized. Displayed in this plot are the data for selective transporters (unconstrained substrate K_D_) and unselective transporters (constrained at K_D_ > .1 mM, 1 mM, and 10 mM). It is evident that at the various [S_out_] tested the selective transporters had larger substrate uptake rates than the unselective ones.

These results confirm that SLCs can raise their transport capacity by becoming more specific for their cognate substrates. We, therefore, consider it plausible that specific SLCs arose from ancestors, which were unselective and that this transformation was driven by the need to support high substrate uptake rates. Conversely, there are transporters, which are under evolutionary pressure to remain unselective, because they support the disposition of xenobiotics. This is exemplified by members of the SLC22 family, which recognize diverse substrates to mediate disposition of drugs and xenobiotics: Both organic cation (OCT1-3/SLC22A1-3) and anion transporters (OAT1-3/SLC22A6-8) translocate most of their substrates with K_M_ values in the high μM range (VanWert et al., 2010; Motohashi and Inui, 2013). This is despite the fact that, in most instances, they are faced with substrate concentrations in the low micromolar range. However, we find this in good agreement with our results, which showed that the unselective transporters that we optimized for 1 μM S_out_, displayed K_Ms_ in the submillimolar range (see Figure 5E). It is worth noting that the SLC22 family also encompasses members, which have a narrow substrate specificity; these have K_M_ value in the low micromolar range.

## Discussion

Solute carriers have a long evolutionary history: More than 50% of SLC subfamilies, which are present in the human genome, are also found in prokaryotes (Hoglund et al., 2011). Eukaryotic transporters have longer N- and C-termini than bacterial transporters. This presumably reflects evolutionary adaptation to the increase in complexity: The N- and C-termini harbor site for posttranslational modifications (e.g., phosphorylation by protein kinases) and docking sites for the protein machinery required for trafficking between cellular compartments (Chiba et al., 2014). The evolutionary history also suggests that individual SLC subfamilies expanded and contracted during phylogenesis (Caveney et al., 2006; Denecke et al., 2020). Expansion was not only driven by the adaptation to new substrates but also by the requirements to optimize concentrative power and uptake rate: Phosphate transporters of the SLC34 subfamily differ in their concentrative power and in their electrogenicity (Forster et al., 2013). Similarly, the three closely related monoamine transporters provide different solution to the trade-off between harvesting the membrane potential and maintaining constant uptake at variable voltage (Bhat et al., 2021). Here, we explored how the concentration of substrate, which a solute carrier encounters on both, the extra- and intracellular side can exert evolutionary pressure on the operating mode of a transporter. Our approach relied on analytical expressions for descriptors of transporter function (i.e., K_M_ and V_max_ of substrate transport) as a function of the microscopic rate constants, which parameterize the kinetic models of SLC. Arguably, the outcome of evolutionary adaptation must maximize substrate uptake rate at the prevailing conditions. Accordingly, our optimization algorithm searched for the microscopic rate constants, which yielded the largest possible value for the substrate uptake rate. The pertinent insights can be summarized as follows: (i) low extracellular substrate concentrations select for transporters, which have low K_M_ and V_max_. Only this combination allows for a high rate in the transport cycle, but there is a surprisingly broad range of microscopic rate constants, which support this solution. (ii) In contrast, a transporter operating at high extracellular substrate concentrations has a substantially more restricted parameter space and maintains a high uptake rate only if it has a high K_M_ and a high V_max_ for substrate. (iii) Random order of substrate and co-substrate binding is superior to all possible sequential orders, if a transporter is to maintain a high rate of substrate uptake in the presence of accumulating intracellular substrate, because it allows for cooperative binding.

**Figure 6:**
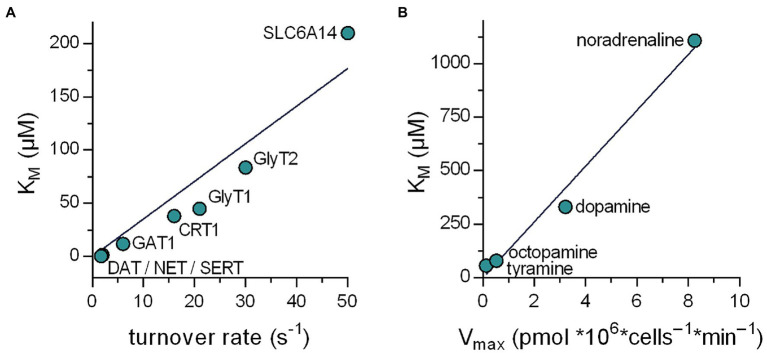
Relation between the K_M_ and the substrate turnover rate of transporters in the SLC6 family. **(A)** Plotted are data obtained from literature of eight members of the SLC6 family. The line in the graph is a linear fit to the data points (*r*^2^ = .915). It is evident that in the real world a positive correlation exists between the K_M_ and the turnover rate. **(B)** Relation between K_M_ and V_max_ for the various substrates of the *Lumbricus terrestris* monoamine transporter. The data were taken from Caveney et al. (2006). The line in the graph is a linear fit to the data points (*r*^2^ = .981).

Solute carriers have long been known to fall into two categories, that is, high-affinity–low-capacity transporters and low affinity–high capacity transporters. It is important to note, however, that there is not any relation between the K_M_ value and the V_max_ value, which *a priori* dictates that these two parameters must move in the same direction. We examined the relation between turnover rates and K_M_ in the SLC6 family, because turnover rates have been determined with high precision by electrophysiological recordings (Bicho and Grewer, 2005; Erdem et al., 2019; Bhat et al., 2021; Shi et al., 2021) and the individual steps of the transport cycle have been analyzed in detail. In addition, the K_M_ values for cognate substrate span more than two orders of magnitude. It is evident from Figure 6A that there is a good correlation (*r*^2^ = .915) between turnover rate and K_M_. Similarly, the monoamine transporter of the earthworm *Lumbricus terrestris* can translocate several substrates albeit with substantial differences in V_max_ and K_M_: The K_M_ for norepinephrine is 20-fold higher than for tyramine (Caveney et al., 2006). Again, there is a remarkable correlation (*r*^2^ = .981) between uptake velocity and K_M_ (Figure 6B). It is, therefore, safe to conclude that the existing dichotomy—in the real world and in our data sets—is a consequence of the optimization of the substrate uptake rates.

Similarly, our optimization algorithm required boundary conditions to identify realistic maxima. Table 2 provides a compilation of substrate turnover rates reported for a collection of SLC. The list includes carriers, which cycle at a rate of 3 s^−1^ as well as such that cycle at a rate of about 700 s^−1^. These rates are reasonably close to those, which the optimization algorithm returned, that is, about 500 s^−1^ and 4 s^−1^ for S_out_ 100 μM and .1 μM, respectively. This confirms that the constraints, which we imposed in the optimization, were realistic. Importantly, our analysis establishes a relation between the turnover rate of the transporter and the substrate concentration for which it was optimized. Thus, the substrate turnover rate allows for inferring the concentration range, in which a candidate solute carrier operates under physiological conditions.

**Table 2:** Substrate turnover rates of transporters from various species.

Transporter	Species	Turnover (s^−1^)	Reference	BNID
Lactose permease (LacY)	*Escherichia col*i	40–60	Wright and Overath, 1984	103159
		21	Smirnova et al., 2011	112482
High-affinity glucose transporter 2 HXT2	*Saccharomyces cerevisiae*	53	Kruckeberg et al., 1999	101739
High-affinity hexose transporter 7 HXT7	*Saccharomyces cerevisiae*	197	Ye et al., 2001	101737
Histidine permease	*Salmonella typhimurium*	2	Nikaido et al., 1997	109030
Na(+)/H(+) exchanger 1 (NHE1)	Chinese hamster	80.3 (22°C) 742 (37°C)	Cavet et al., 1999	105479
Na(+)/H(+) exchanger 2 (NHE2)	Chinese hamster	92.1 (22°C) 459 (37°C)	Cavet et al., 1999	105479
Na(+)/H(+) exchanger 3 (NHE3)	Chinese hamster	99.2 (22°C) 609 (37°C)	Cavet et al., 1999	105479

Values were obtained by searching the BioNumbers database (Milo et al., 2010). The respective entries can be accessed via the given BioNumbers ID (BNID).

We subjected transporters to a selection by optimization at low substrate concentration but at high concentrations of Na^+^ co-substrate Nevertheless, some of the solutions produced hypothetical transporters, which were optimized to operate at very low Na^+^ concentrations (*cf*. Figures 1G,H). While these are obviously of little benefit to a multicellular organism with homeostatic control of extracellular ion composition, these transporters are optimally adapted to support nutrient uptake of a unicellular organism invading ecological niches with low ambient salt concentrations. Bacterial transport is thought to rely mainly on the proton motive force. However, there are several examples of bacterial Na^+^-dependent symporters (Wilson and Ding, 2001). In addition, our analysis showed that K_M_ values for substrate and co-substrate differed substantially from the K_D_ values for their binding to the outward-facing conformation (*cf*. Figures 1F,I). This highlights the fact that experimentally determined K_M_ values for substrate and co-substrate are not necessarily adequate measures of true affinity (i.e., k_off_/k_on_). Transporters are forced to adjust ratios of binding and unbinding reactions, which keep the optimum K_D_ values in a narrow range at both, low and high substrate concentrations. In contrast, the initiation of the transport cycle is limited by the apparent on rates of substrate and the co-substrate ion(s). It is worth noting that all solutions resulted in transporters, which bound substrate at a rate close to the diffusion-imposed limit (*cf*. Table 1). As a consequence, this allows for the emergence of high-affinity/low-capacity SLC, which translocate substrate effectively at very low co-substrate concentrations, because large variations in k_on_ for Na^+^ are tolerated.

Substrate accumulation on the intracellular side also exerts a selective pressure. It is low, if the transporter operates in a relay with another transporter, which sequesters the substrate, or with an enzyme, which modifies the substrate: The monoamine transporters for dopamine (DAT/SLC6A3), norepinephrine (NET/SLC6A2), and (SERT/SLC6A4) need not cope with rising intracellular level of their cognate substrate because of the action of vesicular monoamine transporters (vMAT1/SLC18A1 & vMAT2/SLC18A2) which shuffle cytosolic monoamines into vesicles (Hou and Matherly, 2014; Sitte and Freissmuth, 2015). Similarly, the reduced folate carrier (RFC/SLC19A1) and the proton-coupled folate transporter (PCFT/SLC46A4) are unlikely to face inhibition by accumulation of intracellular folate, because it is converted to polyglutamylated folate by folylpolyglutamate synthase (Raz et al., 2016). In fact, it has been argued that, in several instances, transporters and metabolizing enzymes are spatially organized to promote sustained influx of substrate: Direct or indirect tethering of enzymes to SLCs creates membrane transport metabolons, which effectively lower [S_in_] to preclude inhibition on the intracellular side (Moraes and Reithmeier, 2012). In contrast, the creatine transporter-1 (CrT-1/SLC6A8) must maintain the forward transporter mode, although intracellular concentrations are in the range of 5–7 mM (Wyss and Kaddurah-Daouk, 2000). Our analysis allows for understanding how the adaptation to high intracellular substrate is achieved. It is obvious that the transporter can only progress in the forward cycle mode, if the inward-facing conformation of the transporter has a low affinity for the substrate, because a low affinity precludes rebinding of the substrate. However, to afford a lower substrate affinity to the inward-facing conformation, the K_M_ for substrate must increase and, as a corollary, the true affinities of the substrate and the Na^+^ ions for the outward-facing must decrease. In this context, it is worth mentioning that the three monoamine transporter (NET/SLC6A2, DAT/SLC6A3, and SERT/SLC6A4) display high K_M_ for substrate and Na^+^, that is, in the low micromolar and millimolar range, respectively, (Bulling et al., 2012; Li et al., 2017; Bhat et al., 2021). In contrast, the glycine transporter-1 (GlyT1/SLC6A9) and CrT1/SLC6A8 feature about 3–20-fold lower apparent affinities for their cognate substrate and co-substrate Na^+^ (Boehm et al., 2003; Erdem et al., 2019). This is consistent with the fact that these transporters support influx of their cognate substrates in the presence of millimolar concentrations of S_in_, while the monoamine transporters do not. Thus, the solutions, which were explored by our optimization algorithm, reflect parameter space visited during the evolutionary adaptation of transporters.

Our study has several limitations: (i) We restricted our analysis to sodium symporters with a 2 Na^+^ and 1 substrate stoichiometry. It is evident, however, that the approach can be extended to any other stoichiometry of symport. Our approach is also applicable to antiporters although incorporating the k_on_ and k_off_ of the counter-transported solute substantially increases the parameter space and thus the computational effort. (ii) We did not examine the impact of allosteric regulation, which is a major computational challenge. It is clear, however, that evolution also selected for transporters for allostery: DAT/SLC6A3, for instance, harbors an allosteric Zn^2+^ binding site, while its next relatives NET/SLC6A2 and SERT/SLC6A4 do not (Li et al., 2017). (iii) While modeling the evolutionary trajectory to substrate specificity, we assumed that an ancestral unselective transporter gave rise to a specific transporter. This need not be the case. In fact, two rounds of genome duplication events are thought to have occurred after the split of vertebrates from cephalochordates (Wolfe, 2001; Simakov et al., 2020). Duplicated transporters have two possible evolutionary fates: They can be subject to inactivating mutations and lost. Alternatively, they can accumulate mutations, which initially relax their substrate specificity and eventually evolve by further mutations into transporters with novel substrate specificity. We also did not explore the structural basis for evolution in substrate affinity. It is worth noting that solute carries can achieve the substrate translocation step by three different mechanisms, that is, based on a rocker switch, a rocking bundle and a sliding elevator (Diallinas, 2021). In transporters, which operate *via* a sliding elevator mechanism, the substrate binding site must reside in the elevator, which moves along the scaffold domain in a direction perpendicular to the membrane plane and thus translocates the substrate. Surprisingly, in the uric acid-xanthine permease *UapA* of *Aspergillus nidulans*, which operates *via* a sliding elevator mechanism, selectivity can be relaxed by several mutations, which are in the scaffold domain and thus outside of the binding site proper (Diallinas, 2021). Two non-mutually exclusive interpretations have been provided for the effect of the mutations: They may eliminate a selectivity filter, which restricts access to the binding site (Vlanti et al., 2006), and/or they may relax a brake and thus facilitate triggering of the elevator movement (Diallinas, 2021). In transporters, which operate by a rocker switch or a rocking bundle mechanism, the binding site is more deeply buried in the hydrophobic core of the protein than in those operating as sliding elevators. Accordingly, it is readily conceivable that restrictions can be imposed on access to the binding site. In fact, the selective binding to the closely related DAT/SLC6A3 and SERT/SLC6A4 is determined by the association rate constant rather than the dissociation rate constant (Hasenhuetl et al., 2015); access to the binding site is limited—at least in part—by the extracellular loops (Esendir et al., 2021). Exchanging the N-terminus of DAT/SLC6A3 with that of SERT/SLC6A4 reduces the K_M_ of the resulting chimaera for dopamine (Sweeney et al., 2017). Taken together, these observations suggest that changes in transporter selectivity and uptake rates can be brought about by mutations of many residues, which are not necessarily confined to the substrate binding site. During evolution the sequential impact of mutations is likely to change microscopic rate constants along trajectories as diverse as explored by our computational optimization. A long-term challenge is to delineate such an evolutionary path through the landscape of possible microscopic rate constants by sequential mutagenesis. This should be gratifying, because it is expected shed light on the evolutionary history underlying transporter diversity.

## Data Availability Statement

The original contributions presented in the study are included in the article/Supplementary Material, further inquiries can be directed to the corresponding author.

## Author Contributions

KS, MF, and WS conceptualized and wrote the manuscript. KS scripted the optimization algorithm. CF, DB, KS, and WS analyzed the data. All authors contributed to the article and approved the submitted version.

## Funding

This work was funded by a grant from the Austrian Science Fund/FWF (P31813 to WS) and the Vienna Science and Technology Fund/WWTF (LSC17-026 to MF).

## Conflict of Interest

The authors declare that the research was conducted in the absence of any commercial or financial relationships that could be construed as a potential conflict of interest.

## Publisher’s Note

All claims expressed in this article are solely those of the authors and do not necessarily represent those of their affiliated organizations, or those of the publisher, the editors and the reviewers. Any product that may be evaluated in this article, or claim that may be made by its manufacturer, is not guaranteed or endorsed by the publisher.
