# Supplementary material for: Optimizing the Substrate Uptake Rate of Solute Carriers
Source: Front Physiol. 2022 Feb 3;13:817886. doi: 10.3389/fphys.2022.817886 (PMC8850955; doi:10.3389/fphys.2022.817886)
Supplement: Supplementary file 1 [file Table_1.DOCX]

**Substate Uptake Rate =** (k_3_4*(CNaI^2*CSI*k_1_6*k_2_1^2*k_4_3*k_5_4^2*k_6_5 + CNaO^2*CSO*k_1_2^2*k_2_3*k_4_5^2*k_5_6*k_6_1 + CNaO^2*CSO*k_1_2^2*k_2_3*k_4_3*k_5_6*k_6_1 + CNaI^2*CSI*CSO*k_1_6*k_2_3*k_4_3*k_5_4^2*k_6_5 + CNaI^2*CNaO^2*CSO*k_1_2^2*k_2_3*k_4_3*k_5_4^2*k_6_1 + CNaI^2*CNaO^2*CSI*CSO*k_1_2^2*k_2_3*k_4_3*k_5_4^2*k_6_5))/(k_1_6*k_2_1^2*k_3_2*k_4_5^2*k_5_6 + k_1_6*k_2_1^2*k_3_4*k_4_5^2*k_5_6 + k_2_1^2*k_3_2*k_4_5^2*k_5_6*k_6_1 + k_2_1^2*k_3_4*k_4_5^2*k_5_6*k_6_1 + k_1_6*k_2_1^2*k_3_2*k_4_3*k_5_6 + k_2_1^2*k_3_2*k_4_3*k_5_6*k_6_1 + CSI*k_1_6*k_2_1^2*k_3_2*k_4_3*k_6_5 + CSO*k_1_6*k_2_3*k_3_4*k_4_5^2*k_5_6 + CSO*k_2_3*k_3_4*k_4_5^2*k_5_6*k_6_1 + CNaO^2*k_1_2^2*k_3_2*k_4_3*k_5_6*k_6_1 + CSI*k_1_6*k_2_1^2*k_3_2*k_4_5^2*k_6_5 + CSI*k_1_6*k_2_1^2*k_3_4*k_4_5^2*k_6_5 + CNaI^2*k_1_6*k_2_1^2*k_3_2*k_4_3*k_5_4^2 + CNaI^2*k_2_1^2*k_3_2*k_4_3*k_5_4^2*k_6_1 + CNaO^2*k_1_2^2*k_3_2*k_4_5^2*k_5_6*k_6_1 + CNaO^2*k_1_2^2*k_3_4*k_4_5^2*k_5_6*k_6_1 + CNaI^2*CSI*k_1_6*k_2_1^2*k_3_2*k_5_4^2*k_6_5 + CNaI^2*CSI*k_1_6*k_2_1^2*k_3_4*k_5_4^2*k_6_5 + CNaI^2*CSI*k_1_6*k_2_1^2*k_4_3*k_5_4^2*k_6_5 + CNaI^2*CSI*k_2_1^2*k_3_2*k_4_3*k_5_4^2*k_6_5 + CNaO^2*CSO*k_1_2^2*k_2_3*k_3_4*k_4_5^2*k_5_6 + CNaO^2*CSO*k_1_2^2*k_2_3*k_3_4*k_4_5^2*k_6_1 + CNaO^2*CSO*k_1_2^2*k_2_3*k_4_5^2*k_5_6*k_6_1 + CSI*CSO*k_1_6*k_2_3*k_3_4*k_4_5^2*k_6_5 + CNaI^2*CNaO^2*k_1_2^2*k_3_2*k_4_3*k_5_4^2*k_6_1 + CNaI^2*CSI*k_1_6*k_3_2*k_4_3*k_5_4^2*k_6_5 + CNaO^2*CSO*k_1_2^2*k_2_3*k_3_4*k_5_6*k_6_1 + CNaO^2*CSO*k_1_2^2*k_2_3*k_4_3*k_5_6*k_6_1 + CNaI^2*CSI*CSO*k_1_6*k_2_3*k_3_4*k_5_4^2*k_6_5 + CNaI^2*CSI*CSO*k_1_6*k_2_3*k_4_3*k_5_4^2*k_6_5 + CNaO^2*CSI*CSO*k_1_2^2*k_2_3*k_3_4*k_4_5^2*k_6_5 + CNaI^2*CNaO^2*CSI*k_1_2^2*k_3_2*k_4_3*k_5_4^2*k_6_5 + CNaI^2*CNaO^2*CSO*k_1_2^2*k_2_3*k_3_4*k_5_4^2*k_6_1 + CNaI^2*CNaO^2*CSO*k_1_2^2*k_2_3*k_4_3*k_5_4^2*k_6_1 + CNaI^2*CNaO^2*CSI*CSO*k_1_2^2*k_2_3*k_3_4*k_5_4^2*k_6_5 + CNaI^2*CNaO^2*CSI*CSO*k_1_2^2*k_2_3*k_4_3*k_5_4^2*k_6_5) - (k_4_3*(CNaI^2*CSI*k_1_6*k_2_1^2*k_3_2*k_5_4^2*k_6_5 + CNaI^2*CSI*k_1_6*k_2_1^2*k_3_4*k_5_4^2*k_6_5 + CNaO^2*CSO*k_1_2^2*k_2_3*k_3_4*k_5_6*k_6_1 + CNaI^2*CSI*CSO*k_1_6*k_2_3*k_3_4*k_5_4^2*k_6_5 + CNaI^2*CNaO^2*CSO*k_1_2^2*k_2_3*k_3_4*k_5_4^2*k_6_1 + CNaI^2*CNaO^2*CSI*CSO*k_1_2^2*k_2_3*k_3_4*k_5_4^2*k_6_5))/(k_1_6*k_2_1^2*k_3_2*k_4_5^2*k_5_6 + k_1_6*k_2_1^2*k_3_4*k_4_5^2*k_5_6 + k_2_1^2*k_3_2*k_4_5^2*k_5_6*k_6_1 + k_2_1^2*k_3_4*k_4_5^2*k_5_6*k_6_1 + k_1_6*k_2_1^2*k_3_2*k_4_3*k_5_6 + k_2_1^2*k_3_2*k_4_3*k_5_6*k_6_1 + CSI*k_1_6*k_2_1^2*k_3_2*k_4_3*k_6_5 + CSO*k_1_6*k_2_3*k_3_4*k_4_5^2*k_5_6 + CSO*k_2_3*k_3_4*k_4_5^2*k_5_6*k_6_1 + CNaO^2*k_1_2^2*k_3_2*k_4_3*k_5_6*k_6_1 + CSI*k_1_6*k_2_1^2*k_3_2*k_4_5^2*k_6_5 + CSI*k_1_6*k_2_1^2*k_3_4*k_4_5^2*k_6_5 + CNaI^2*k_1_6*k_2_1^2*k_3_2*k_4_3*k_5_4^2 + CNaI^2*k_2_1^2*k_3_2*k_4_3*k_5_4^2*k_6_1 + CNaO^2*k_1_2^2*k_3_2*k_4_5^2*k_5_6*k_6_1 + CNaO^2*k_1_2^2*k_3_4*k_4_5^2*k_5_6*k_6_1 + CNaI^2*CSI*k_1_6*k_2_1^2*k_3_2*k_5_4^2*k_6_5 + CNaI^2*CSI*k_1_6*k_2_1^2*k_3_4*k_5_4^2*k_6_5 + CNaI^2*CSI*k_1_6*k_2_1^2*k_4_3*k_5_4^2*k_6_5 + CNaI^2*CSI*k_2_1^2*k_3_2*k_4_3*k_5_4^2*k_6_5 + CNaO^2*CSO*k_1_2^2*k_2_3*k_3_4*k_4_5^2*k_5_6 + CNaO^2*CSO*k_1_2^2*k_2_3*k_3_4*k_4_5^2*k_6_1 + CNaO^2*CSO*k_1_2^2*k_2_3*k_4_5^2*k_5_6*k_6_1 + CSI*CSO*k_1_6*k_2_3*k_3_4*k_4_5^2*k_6_5 + CNaI^2*CNaO^2*k_1_2^2*k_3_2*k_4_3*k_5_4^2*k_6_1 + CNaI^2*CSI*k_1_6*k_3_2*k_4_3*k_5_4^2*k_6_5 + CNaO^2*CSO*k_1_2^2*k_2_3*k_3_4*k_5_6*k_6_1 + CNaO^2*CSO*k_1_2^2*k_2_3*k_4_3*k_5_6*k_6_1 + CNaI^2*CSI*CSO*k_1_6*k_2_3*k_3_4*k_5_4^2*k_6_5 + CNaI^2*CSI*CSO*k_1_6*k_2_3*k_4_3*k_5_4^2*k_6_5 + CNaO^2*CSI*CSO*k_1_2^2*k_2_3*k_3_4*k_4_5^2*k_6_5 + CNaI^2*CNaO^2*CSI*k_1_2^2*k_3_2*k_4_3*k_5_4^2*k_6_5 + CNaI^2*CNaO^2*CSO*k_1_2^2*k_2_3*k_3_4*k_5_4^2*k_6_1 + CNaI^2*CNaO^2*CSO*k_1_2^2*k_2_3*k_4_3*k_5_4^2*k_6_1 + CNaI^2*CNaO^2*CSI*CSO*k_1_2^2*k_2_3*k_3_4*k_5_4^2*k_6_5 + CNaI^2*CNaO^2*CSI*CSO*k_1_2^2*k_2_3*k_4_3*k_5_4^2*k_6_5)
